# Draft Genome Sequence of Pediatric Otitis Media Isolate Streptococcus pneumoniae Strain EF3030, Which Forms *In Vitro* Biofilms That Closely Mimic *In Vivo* Biofilms

**DOI:** 10.1128/MRA.01114-18

**Published:** 2019-01-10

**Authors:** Edgar J. Scott, Nicole R. Luke-Marshall, Anthony A. Campagnari, David W. Dyer

**Affiliations:** a Department of Microbiology and Immunology, University of Oklahoma Health Sciences Center, Oklahoma City, Oklahoma, USA; b Department of Microbiology and Immunology, State University of New York at Buffalo, Buffalo, New York, USA; University of California, Riverside

## Abstract

Here, we report the draft genome sequence of Streptococcus pneumoniae EF3030, a pediatric otitis media isolate active in biofilm assays of epithelial colonization. The final draft assembly included 2,209,198 bp; the annotation predicted 2,120 coding DNA sequences (CDSs), 4 complete rRNA operons, 58 tRNAs, 3 noncoding RNAs (ncRNAs), and 199 pseudogenes.

## ANNOUNCEMENT

Streptococcus pneumoniae (the pneumococcus), an encapsulated Gram-positive diplococcus, is one of the most common colonizers and opportunistic pathogens of the human upper respiratory tract. It is estimated that 95% of children by the age of two are colonized on the nasopharyngeal mucosa by at least 1 of over 90 S. pneumoniae serotypes, which persist asymptomatically in healthy individuals into adulthood (1–4). S. pneumoniae is the primary etiologic agent of otitis media and secondary bacterial pneumonia following viral infection and causes severe invasive disease, including acute pneumonia, meningitis, and sepsis (2, 5). Pneumococcal biofilm formation *in vivo* contributes to immune evasion, antibiotic resistance, and persistence and serves as a reservoir for initiating local and invasive disease (recently reviewed in reference 6). S. pneumoniae strain EF3030, a capsular serotype 19F, is a pediatric otitis media isolate that is an efficient colonizer of murine model systems (6–11). In addition, *in vitro* S. pneumoniae EF3030 biofilms on an epithelial substratum closely mimic *in vivo* biofilms that form during asymptomatic colonization (6, 8). On the human upper respiratory mucosa, polymicrobial interactions within the microbiome likely impact the mechanisms of disease induction by the pneumococcus and other cocolonizing microbes. Studies designed to delineate these complex interactions are warranted and the whole-genome sequence of S. pneumoniae EF3030 will contribute to the identification and characterization of bacterial factors critical for these processes.

Whole-genome sequencing was performed on an Illumina MiSeq instrument, which generated 1,646,744 paired-end reads, with an average read length of 151 bp (219× coverage). An initial reference assembly of the paired-end reads was first performed against S. pneumoniae R6, from which multilocus sequence type (MLST) (12) loci (*aroE*, *gdh*, *gki*, *recP*, *spi*, *xpt*, and *ddl*) were extracted. These loci were then used in an MLST comparison to the available completed S. pneumoniae genomes, from which we determined that S. pneumoniae CGSP14 (GenBank accession number CP001033) was the most closely related strain. The raw S. pneumoniae EF3030 reads were then reassembled to the S. pneumoniae CGSP14 genome, using Bowtie 2 with the very sensitive local preset (13), SAMtools (14), BCFtools (15), and vcfutils (16). The exact code for this assembly was “samtools mpileup -uf ./spn_cgsp14.fna sorted.bam | bcftools call -c | vcfutils.pl vcf2fq > cns.fq.” An additional *de novo* assembly employed SOAPdenovo2 (kmer size, 80) (17) and identified seven protein-coding contigs not in the reference assembly. These were all homologous to previously identified S. pneumoniae genes and consequently were already contained in the S. pneumoniae pan-genome (18). These data were submitted to the NCBI Prokaryotic Genome Annotation Pipeline (19) for annotation. The annotation consisted of 2,193 total genes, including 2,120 coding DNA sequences (CDSs), 73 RNA-encoding genes (4 complete rRNA operons, 58 tRNAs, and 3 noncoding RNAs [ncRNAs]), and 199 pseudogenes.

### Data availability.

The draft genome sequence has been deposited in NCBI GenBank under the accession number CP026549. The raw data were deposited in the Sequence Read Archive under BioProject accession number PRJNA432428.
